# Selective dorsal rhizotomy using a 3D high definition exoscope

**DOI:** 10.3171/2023.10.FOCVID23105

**Published:** 2024-01-01

**Authors:** Jia Xu Lim, Wan Tew Seow, Zhi Min Ng, Sharon Y. Y. Low

**Affiliations:** 1 Neurosurgical Service, KK Women’s and Children’s Hospital, Singapore;; 2 Department of Neurosurgery, National Neuroscience Institute, Singapore;; 3 SingHealth-Duke NUS Neuroscience Academic Clinical Programme, Singapore;; 4 Neurology Service, Department of Pediatrics, KK Women’s and Children’s Hospital, Singapore; and; 5 SingHealth-Duke NUS Pediatrics Academic Clinical Programme, Singapore

**Keywords:** exoscope, selective dorsal rhizotomy, spastic diplegia

## Abstract

Selective dorsal rhizotomy (SDR) is an established neurosurgical technique for children with spastic diplegia secondary to cerebral palsy. Meticulous intraoperative testing of individual nerve roots with electromyography in tandem with the on-site neurorehabilitation team is recommended for good clinical outcomes. The standard approach requires the neurosurgeons to spend extended time under the traditional operating microscope. In this video, the authors describe the use of a 3D exoscope system for SDR. Overall, the 3D exoscope improves ergonomics and reduces musculoskeletal fatigue for the operating neurosurgeons. Furthermore, it provides excellent visualization of important structures, allowing safe and efficient completion of the procedure.

The video can be found here: https://stream.cadmore.media/r10.3171/2023.10.FOCVID23105

## Transcript

This presentation provides an overview of our experience in performing SDR using a high-definition 3D exoscope system. Here are the abbreviations that we have used for this presentation.

### 0:33 Patient Description.

Our patient was a 6-year-old male with a background of cerebral palsy with spastic diplegia. Clinically, he was Gross Motor Function Classification System (GMFCS) level III to IV and had a modified Ashworth Scale of 3 involving bilateral lower limbs. There was no dystonia or ataxia. Selective dorsal rhizotomy was decided as the procedure of choice after a multidisciplinary team (MDT) consensus. The MDT consists of neurosurgeons, orthopedic surgeons, neurorehabilitation physicians, and allied health professionals. Preoperative workup as per institutional protocol included thorough neuroimaging, physical and cognitive assessments, followed by a trial period of oral baclofen and targeted areas of botulinum toxin injections in tandem with intensive physiotherapy. As part of this workup, a progressive cerebral pathology was excluded.

### 1:26 Surgical Considerations.

The aim of SDR is to reduce spasticity while ensuring adequate tone and power is preserved for long-term motor function, especially ambulation.^1^ In the context of our case, the surgery is performed with the assistance of a high-definition 3D exoscope. We are using the Synaptive Modus V exoscope.

### 1:48 High-Definition 3D Exoscope.

An exoscope confers significant advantages in terms of surgical visualization and ergonomics.^2–4^ In recent years, there is a growing body of literature with regard to its use in neurosurgery, especially in adult spine conditions.^4,5^ Separately, SDR is a procedure that may be prolonged owing to time spent on intraoperative electromyography testing of individual nerve roots in tandem with the neurorehabilitation team’s assessment. This aspect of the surgery is extremely important to achieve the optimal reduction in limb spasticity. Under such circumstances, the 3D exoscope allows the neurosurgical team to operate in a more comfortable posture and, at the same time, provide excellent visualization for both the surgeons as well as the neurorehabilitation team. Nonetheless, the setup for the 3D exoscope system tends to cause crowding of operating space.^2,3^ This drawback becomes relevant for SDR, where manpower, operating tools, and neuromonitoring equipment need to share limited space with the 3D exoscope setup. Therefore, strategic rationalization of space is paramount and will be subsequently discussed.

### 3:00 Preparation.

Here, the neurosurgical team flanks the patient and the scrub team is placed opposite to the lead neurosurgeon. We placed the anesthesia team and their equipment at the head of the operating table, allowing the neurorehabilitation team at the opposite end to access the lower limbs freely when needed. The 3D exoscope is situated beside the lead neurosurgeon, and both large screens are placed at an angle where both the lead and assistant surgeons are able to comfortably view with minimal neck movement. All present at the surgery are provided with 3D glasses to view the surgery in real time.

### 3:34 Positioning.

After general anesthesia, the patient is placed in a prone position for lumbar spine surgery with relevant pressure areas protected. Intraoperative neurophysiological monitoring electrodes for motor evoked potentials, somatosensory evoked potentials, and electromyography (EMG) are placed accordingly. Next, the patient is surgically draped in a manner that allows the neurorehabilitation team to test the lower limbs intraoperatively.

### 4:01 Procedure.

After skin exposure of the lumbar spine, a standard laminotomy from L1 to S1 was performed. Surgical exposure was further optimized with Kerrison rongers. A pointer wirelessly linked to the 3D exoscope was used to optimize visualization. Next, durotomy was performed via a long midline incision. Tacking sutures are placed. The view is oriented with the right screen being cranial, left screen being caudal, and the top being the patient’s left. The nerve root bundles of the cauda equina were separated and traced back to their relevant exit foramina. Individual roots were subsequently separated further into dorsal sensory and ventral motor root components. Confirmation of their function was tested with EMG. Each nerve root bundle was then isolated with surgical patties. This process was meticulously repeated for each nerve root bundle. Following that, the sensory roots were then split into its rootlets. At this point, the rootlets were stimulated once more for reconfirmation prior to sectioning. During this process, the neurorehabilitation team begins to assess for the reduction of spasticity. This will be repeated for each dorsal root. As demonstrated, the 3D exoscope was able to produce superb visuals while reducing surgical fatigue of the surgeons during this long procedure. An additional advantage was the system’s pointer that was able to further enhance this by guiding the exoscope into position and thus ensuring an optimal angle of view.

### 6:21 Closure.

Closure is performed in layers following the principles of previously published literature.^6^

### 6:26 Reflections.

Benefits of this system include excellent intraoperative visualization, improved ergomics, and the reduction of musculoskeletal fatigue in a prolonged surgery. Its high magnification and illumination views offer a wide operative corridor, increased focal length range and depth, and immersive surgical experience.^7^ The drawbacks of theater crowding can be overcome with conscientious preoperative planning and coordination with the different subspecialty teams involved.

### 6:55 Future Directions.

Presently, the operating microscope remains the mainstay of most neurosurgical procedures, and its ease of setup has been instilled at our institution for several years. Under such circumstances, the introduction of the 3D surgical exoscope with its different setup requires a trial period for the surgical team to overcome learning curves.^7^ At the time of this study, we believe its utility may not be suitable for a direct comparison with the established workflow of our operating microscope. In addition, there is no evidence that single-level laminectomy SDR via a smaller incision is superior to a traditional, open laminoplasty technique as described by us.^8^ However, we are keen to maximize the advantage of the 3D surgical exoscope’s excellent magnification by modifying our surgical approach via a smaller incision, and look forward to reducing operative times as part of our future work.^8,9^ To our knowledge, this is the first reported case of the utility of a 3D exoscope system for SDR in a pediatric patient. Our references are presented within.

## Disclosures

The authors report no conflict of interest concerning the materials or methods used in this study or the findings specified in this publication.

## Author Contributions

Primary surgeon: Low, Seow. Editing and drafting the video and abstract: Low, Lim. Critically revising the work: Low, Lim, Ng. Reviewed submitted version of the work: Low, Lim, Ng. Approved the final version of the work on behalf of all authors: Low. Supervision: Low.

## Supplemental Information

### Patient Informed Consent

The necessary patient informed consent was obtained in this study.

## References

[1] Steinbok P Selective dorsal rhizotomy for spastic cerebral palsy: a review Childs Nerv Syst 2007 23 9 981 990 17551739 10.1007/s00381-007-0379-5

[2] Ricciardi L Chaichana KL Cardia A The exoscope in neurosurgery: an innovative "point of view". A systematic review of the technical, surgical, and educational aspects World Neurosurg 2019 124 136 144 30660891 10.1016/j.wneu.2018.12.202

[3] Fiani B Jarrah R Griepp DW Adukuzhiyil J The role of 3D exoscope systems in neurosurgery: an optical innovation Cureus 2021 13 6 e15878 34327102 10.7759/cureus.15878 PMC8302823

[4] Montemurro N Scerrati A Ricciardi L Trevisi G The exoscope in neurosurgery: an overview of the current literature of intraoperative use in brain and spine surgery J Clin Med 2021 11 1 223 35011964 10.3390/jcm11010223 PMC8745525

[5] Lin H Chen F Lin T Beyond magnification and illumination: ergonomics with a 3D exoscope in lumbar spine microsurgery to reduce musculoskeletal injuries Orthop Surg 2023 15 6 1556 1563 37154147 10.1111/os.13737 PMC10235180

[6] Lim JX Low SY Ng LP Seow WT Prevention and treatment of CSF leaks in congenital complex spinal lipomas Acta Neurochir (Wien) 2022 164 4 1157 1160 35015155 10.1007/s00701-021-05095-5

[7] Raheja A Mishra S Garg K Impact of different visualization devices on accuracy, efficiency, and dexterity in neurosurgery: a laboratory investigation Neurosurg Focus 2021 50 1 E18 10.3171/2020.10.FOCUS20786 33386021

[8] Ou C Kent S Miller S Steinbok P Selective dorsal rhizotomy in children: comparison of outcomes after single-level versus multi-level laminectomy technique Can J Neurosci Nurs 2010 32 3 17 24 20865831

[9] Park TS Johnston JM Surgical techniques of selective dorsal rhizotomy for spastic cerebral palsy Technical note Neurosurg Focus 2006 21 2 e7 16918228

